# Feasibility of task-sharing with community health workers for the identification, emergency management and referral of women with pre-eclampsia, in Mozambique

**DOI:** 10.1186/s12978-021-01192-x

**Published:** 2021-07-06

**Authors:** Esperança Sevene, Helena Boene, Marianne Vidler, Anifa Valá, Salésio Macuacua, Orvalho Augusto, Quinhas Fernandes, Cassimo Bique, Eusébio Macete, Mohsin Sidat, Peter von Dadelszen, Khátia Munguambe, CLIP Feasibility Working Group, Rosa Pires, Zefanias Nhamirre, Rogério Chiaú, Analisa Matavele, Adérito Tembe, Lina Machai, Beth Payne, Sharla Drebit, Chirag Kariya, Laura Magee

**Affiliations:** 1 Maternal Health Unit, Centro de Investigação em Saúde da Manhiça (CISM), Manhiça, Mozambique; 2 Department of Physiologic Science, Clinical Pharmacology, Faculty of Medicine, Universidade Eduardo Mondlane, Maputo, Mozambique; 3 Department of Obstetrics and Gynaecology, and The Child and Family Research Unit, University of British Columbia, Vancouver, BC Canada; 4 Department of Community Health, Faculty of Medicine,, Universidade Eduardo Mondlane, Maputo, Mozambique; 5 Ministério da Saúde, Maputo, Mozambique; 6 Hospital Central de Maputo, Maputo, Mozambique; 7 King’s College London, London, UK

**Keywords:** Task-sharing, Community health workers, Pre-eclampsia, Maternal mortality, Mozambique

## Abstract

**Background:**

Maternal mortality is an important public health problem in low-income countries. Delays in reaching health facilities and insufficient health care professionals call for innovative community-level solutions. There is limited evidence on the role of community health workers in the management of pregnancy complications. This study aimed to describe the feasibility of task-sharing the initial screening and initiation of obstetric emergency care for pre-eclampsia/eclampsia from the primary healthcare providers to community health workers in Mozambique and document healthcare facility preparedness to respond to referrals.

**Method:**

The study took place in Maputo and Gaza Provinces in southern Mozambique and aimed to inform the Community-Level Interventions for Pre-eclampsia (CLIP) cluster randomized controlled trial. This was a mixed-methods study. The quantitative data was collected through self-administered questionnaires completed by community health workers and a health facility survey; this data was analysed using Stata v13. The qualitative data was collected through focus group discussions and in-depth interviews with various community groups, health care providers, and policymakers. All discussions were audio-recorded and transcribed verbatim prior to thematic analysis using QSR NVivo 10. Data collection was complemented by reviewing existing documents regarding maternal health and community health worker policies, guidelines, reports and manuals.

**Results:**

Community health workers in Mozambique were trained to identify the basic danger signs of pregnancy; however, they have not been trained to manage obstetric emergencies. Furthermore, barriers at health facilities were identified, including lack of equipment, shortage of supervisors, and irregular drug availability. All primary and the majority of secondary-level facilities (57%) do not provide blood transfusions or have surgical capacity, and thus such cases must be referred to the tertiary-level. Although most healthcare facilities (96%) had access to an ambulance for referrals, no transport was available from the community to the healthcare facility.

**Conclusions:**

This study showed that task-sharing for screening and pre-referral management of pre-eclampsia and eclampsia were deemed feasible and acceptable at the community-level, but an effort should be in place to address challenges at the health system level.

## Plain Language Summary

Maternal mortality is an important public health problem in Mozambique. Delays in reaching health facilities and insufficient health care professionals call for innovative community-level solutions. We conducted a study to describe the feasibility of task-sharing the screening and initiation of management for pre-eclampsia/eclampsia from the primary healthcare providers to community health workers in Mozambique and to document healthcare facility preparedness to respond to referrals. The study was done to inform a future intervention trial known as the Community-Level Interventions for Pre-eclampsia (CLIP) study. We interviewed community health workers, women, various community groups, health care providers, and policymakers and assessed health facilities in Maputo and Gaza provinces, Mozambique. Our results showed that community health workers in Mozambique were trained to identify the basic danger signs of pregnancy; however, they were not trained or equipped to provide obstetric emergencies care prior to referral. Nurses at primary health facilities were supportive of task-sharing with community health workers; however, some barriers mentioned include a lack of equipment, shortage of supervisors, and irregular drug availability. Local stakeholders emphasized the need for comprehensive training and supervision of community health workers to take on new tasks. Task-sharing for screening and pre-referral management of pre-eclampsia and eclampsia was deemed feasible at the community level in southern Mozambique, but still, to be addressed some health system level barriers to the management of pregnancies complications.

## Introduction

Maternal mortality is an important public health problem in low- and middle-income countries (LMICs). Unfortunately, the Millennium Development Goal of reducing maternal mortality rate by three-quarters by 2015 [1] was far from being achieved in most low-income countries, where the rates are 100 times higher than those in high-income countries [2]. Countries need to accelerate their strategies to achieve the target of reducing the global maternal mortality ratio to less than 70 per 100,000 live births by 2030, as planned in the Sustainable Development Goal [3].

In Mozambique, the 2007 demographic census showed a maternal mortality rate of 500/100,000 live births with large differences between the provinces (272-822/100,000 live births) [4]. However, despite many efforts to reduce maternal mortality, it has made little progress at 480/100,000 live births in 2015 [2] and 452/100,000 live births in 2017 [5]. Most maternal deaths in Mozambique result from direct causes such as postpartum haemorrhage, hypertension, eclampsia and puerperal sepsis and pre-eclampsia and eclampsia are the top three causes of maternal death [6–8]. There is robust evidence for magnesium sulphate (MgSO_4_) efficacy in the treatment of eclampsia [9–12]. Many more women could benefit from MgSO_4_ if appropriate policies were in place. However, the shortage of health professionals often prevents timely detection and management of hypertension in pregnancy [13]. On the other hand, delays in arriving at health facilities due to long distances, poor road conditions, and the absence of regular transport further complicate maternal care [14–16]. Therefore, new and innovative strategies are needed to improve maternal health outcomes across the country. Strategies should start at the community-level to reduce these delays and the associated maternal and perinatal complications. These strategies may include a package of evidence-based interventions at the community and primary health care (PHC) to identify and rapidly manage pre-eclampsia/eclampsia (PE/E). Evidence-based interventions should include validated methods to predict adverse pregnancy outcomes to allow prompt referral and the initiation of life-saving treatments [17]. Community health workers (CHWs) may be the appropriate community-level cadre to fulfil this task.

CHWs in Mozambique have increasingly taken on clinical tasks, such as the management of malaria, diarrhoea, pneumonia, severe acute malnutrition and home visits for new-borns [18, 19]. Recently the World Health Organization (WHO) and the International Federation of Gynaecology and Obstetrics (FIGO) recognized the need to introduce misoprostol at the community level to prevent post-partum haemorrhage [20]. As a result, some low-income countries, including Mozambique, are adapting their programs to include this recommendation with varying levels of success [18, 21, 22].

This study aimed to describe the feasibility of task-sharing screening and initiation of emergency management of pre-eclampsia between CHWs and primary healthcare providers in Mozambique and to document facility preparedness to respond.

## Methods

### Study design

The findings hereby reported are part of a broader multicentre study on “The Feasibility of Community-Level Interventions for the prevention and treatment of Pre-eclampsia and Eclampsia in selected rural communities of southern Mozambique (CLIP Feasibility)” [23]. The feasibility study was implemented in advance of the CLIP trial (NCT01911494), which aimed to assess a community-level intervention for the management of PE/E. The CLIP trial intervention consisted of community engagement, early identification of danger signs of PE/E, administration of oral methyldopa and intramuscular MgSO_4_ when needed and referral to the nearest health facility. To detect the signs of PE/E, CHWs were equipped with a mHealth application for guided risk stratification. The proposed CLIP Trial intervention that this study aimed to inform was planned to cover two delays, namely seeking care and prompt referral [24, 25].

The formative research study used a mixed methods (qualitative and quantitative). The qualitative approach was based on focus group discussions with health care providers (maternal and child health nurses, midwives, matrons, and TBAs) and other community members (pregnant women, partners and husbands, mothers and mothers-in-law), in-depth interviews with CHW supervisors, the chief medical officers and gynaecologists and obstetricians’ experts. The quantitative data was captured through health facility assessment and community health worker self-administered questionnaires. Data was complemented by a national-level document review of CHWs training curricula, job descriptions, and practice guidelines for the management of pre-eclampsia.

Data collections guides were developed centrally by the study coordination team and adapted from those used in the other countries involved in the study (Nigeria, India and Pakistan). A detailed description of these methods is presented elsewhere [23].

### Study area and population

Mozambique is a low-income country in southern Africa, covering 799,380 Km^2^, with a population of 27,909,798 in 2017 [5]. The GDP is 646 USD per capita, with around 60% of the population living under the poverty line [4]. The country is divided into 11 provinces, which are the basis of the health services administration. The National Health Service (NHS) is based on primary health care, with a 50–60% population coverage rate. The uncovered population live in remote areas with limited access; many of these regions do not have primary health care (PHC). In regions with PHCs, they often do not have adequate human resources, equipment or medicines [26]. In 2019, the total number of medical doctors in the NHS was 2556 (8.7 medical doctors per 100,000 inhabitants), and there were over 6000 maternal and child health nurses (52.6 nurses per 100,000 inhabitants) [27]. To overcome the shortage of medical doctors in the country, nurses and clinical officers have been trained to take on additional duties [28]. In 1978 the country introduced CHWs, designated as *Agentes Polivalentes Elementares* (APEs). CHW are considered volunteers supported by the Ministry of Health (MoH) although they receive a monthly subsidy equivalent to 20 US dollars (1200 meticais) per month as compensation for their work [29, 30]. These workers are chosen by the community and must have basic literacy and arithmetic skills. They complete intensive four-month training. Their responsibilities focus on health promotion and prevention, but in 2010 the MoH revitalized the programme to include some curative activities. Therefore, CHW tasks are continuously being reviewed and adapted [31]. The curative activities focus on the most prevalent childhood illnesses, including malaria, diarrhoea, acute respiratory tract infection, severe acute malnutrition, and home visits for new-borns. In addition, they may promote attendance for antenatal care, identify danger signs in pregnancy and refer pregnant women as needed. However, there is no specific guideline regarding the diagnosis or management of PE/E.

Although in Mozambique, APEs are the leading cadre of CHWs, others also provide obstetric care at the community level, namely the matrons and traditional birth attendants (TBA). Matrons and TBAs are knowledgeable elders in their community with vast experience in maternity care. In Mozambique, the shortage of skilled birth attendants and limited coverage of births in facilities resulted in government support of TBAs and matrons in the late 1980s. However, more recently, the Ministry of Health has discouraged deliveries by TBAs as they were seen to challenge facility-based delivery. There were also concerns regarding the risk of HIV infection due to TBAs poor work conditions [32].

**Fig. 1:**
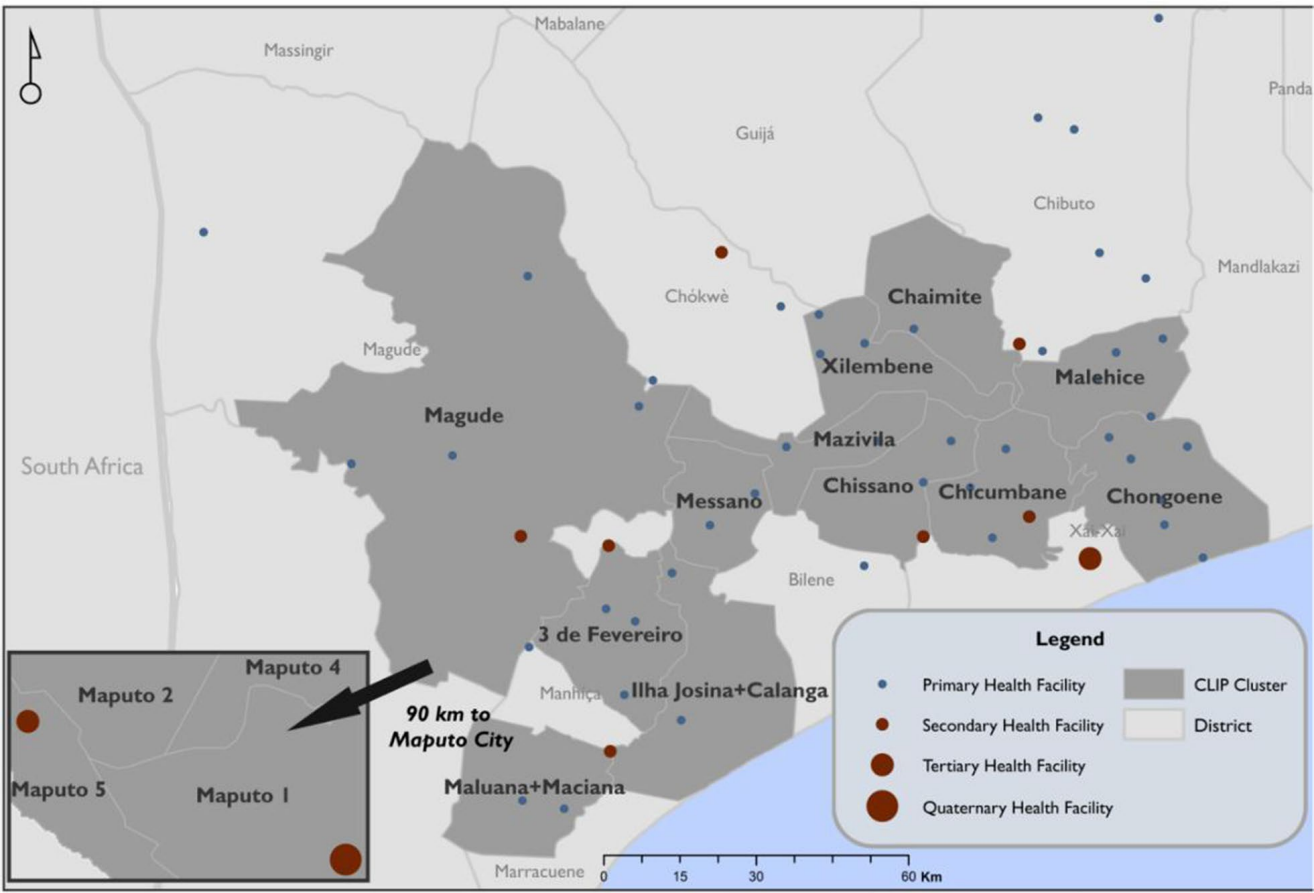
Map of the study area with representation of the health facilities participating in the study

This study was conducted in the two southern-most provinces of Mozambique, namely Maputo and Gaza. In Maputo Province, two districts were involved Manhiça and Magude, while in Gaza, four districts were included Chokwé, Xai-Xai, Chibuto and Macia (Fig. 1). The study area has urban and rural areas. Most residents of this region belong to the *Changana* ethnic group. The predominant occupation is subsistence farming, especially among women. In southern Mozambique, most men migrate to South Africa, Swaziland and other cities in Mozambique for work [4]. Literacy rates vary between the two provinces, with a 22% literacy rate in Maputo and 38% in Gaza; in both cases, literacy is lowest among women [4]. More details on the study site were described elsewhere [33]. This analysis was complemented by a national-level document review of CHWs curricula, job descriptions, and national practice guidelines for the management of pre-eclampsia [34].

### Data collection

This analysis is the triangulation of five sources: a document review, CHW self-administered questionnaires, health facility assessments, in-depth interviews, and focus group discussions. Data collection was conducted between September 2013 and May 2014. This process was conducted by a Mozambican team comprising a junior social scientist (HB), clinicians (AV, AN) and trained interviewers (RP, AM, AT, LM), all employed by the Manhiça Health Research Centre (CISM). The team was supervised by a senior social scientist (KM) and the study principal investigator (ES). Interviewers were selected due to their familiarity with the communities and their research experience. Data collection teams included men and women, fluent in Portuguese and *Changana* (the predominant local language), and had no prior relationship with participants.

### Quantitative data collection

#### Health facility assessment

The health facility questionnaire collected information about the level of care, available medication and commodities, diagnostic capacity, human resource capacity, obstetric statistics, including patient volume, mode of delivery, morbidity and mortality rates. All (56) health facilities in the study area, based on the list of facilities obtained from the Provincial Directorate for Health, were included in this assessment. The health facility questionnaire was completed by the chief nurse in the maternal and child health care. In primary health centres, only one nurse responded, but at other centres, the chief nurse and the midwife responsible for the maternity ward completed the questionnaire. The health facility clinical chief supported the study team in obtaining access to the necessary individuals for interview. The interviews took 30–45 min and were completed when the nurse’s workload was small. The questionnaire comprised of closed-ended questions which were mediated by a trained clinician. Items related to equipment and medications were verified through spot-check observations; only equipment that was physically seen by the study team was considered available.

#### CHW questionnaire

The CHW questionnaire assessed CHW preparedness, knowledge and skills to perform home-based treatment for PE/E and the capacity to give intramuscular injections to pregnant women [35]. Most questions used a Likert-scale and one open-ended question. All CHWs from the study area were included and were reached through the health facility to which they report according to a list provided by the district CHW program focal person. Data collection was conducted either in the health facility where each CHW was registered or at their home. Depending on the number of CHWs, individual or collective briefing sessions by a study team member were given to explain the questionnaire. Because CHWs had limited literacy, a study team member was present at the interview for clarification. The questionnaire took 30–45 min.

### Qualitative data collection

#### In-depth interviews

The interviews focused on the experience of CHW program coordinators regarding CHW requirements, roles and responsibilities, including in the management of pregnant complications. The Mozambican Gynaecologists and Obstetricians Association (AMOG) identified individuals for interview based on their experience and contribution to relevant maternal health policies. At the district level, the chief medical officers and the CHW supervisors were interviewed. The interviews were conducted by trained social scientists. Each interview lasted 30–45 min, was audio-recorded, and notes were taken; they were conducted one-on-one in the house or workplace of participants, depending on their preference.

#### Focus group discussions

The focus group discussion (FGD) guide touched upon the following topics: views on pregnancy complications, pregnancy management, preventive and treatment practices and the health workers' role in managing pregnancy complications. Focus groups were chosen to best capture views of maternal and child health nurses, midwives, matrons and traditional birth attendants, and other community members (pregnant women, partners and husbands, mothers and mothers-in-law). FGDs with nurses and midwives were conducted at health facilities, as these groups could easily be convened and were scheduled at times when health care providers were less busy. For matrons and traditional birth attendants, the FDGs were in *Changana* (local language) and took place at the community, either at the *círculos* (the usual community gathering location) or at the community leaders’ house, according to the groups´ convenience. The FGDs were conducted by trained social scientists. Each FGD lasted between 30 and 80 min, was audio-recorded and notes were also taken.

Panel discussions were also held at the Ministry of Health (with the Deputy Director of Public Health and his team), Maputo Provincial Directorate (with Provincial Medical Chief and his team) and in Manhiça (with members of Community Advisor Committee and with members of CISM Institutional Ethics Review Board). This exercise obtained views regarding task-sharing and was used to understand the policies and the processes related to CHWs.

### Data management and analysis

Information obtained through the desk review was summarized to extract relevant information regarding the history, role and challenges facing CHWs providing maternal and child health care.

The data captured were sent to CISM Data Centre for double data entry and management using REDCap [36]. Prior to sending data to the CISM Data Centre, the study team reviewed each questionnaire in the field. The failures to validation rules and double data entry discrepancies were cross-checked with paper forms for confirmation. Frequency and cross tables were employed for data consistency checks. Outliers and missing values were also reviewed. Data were exported to Stata 13 (Stata Corp., College Station, Texas, USA) for analysis. Frequencies, means, medians, SD and IQR, were used to describe the data.

**Fig. 2:**
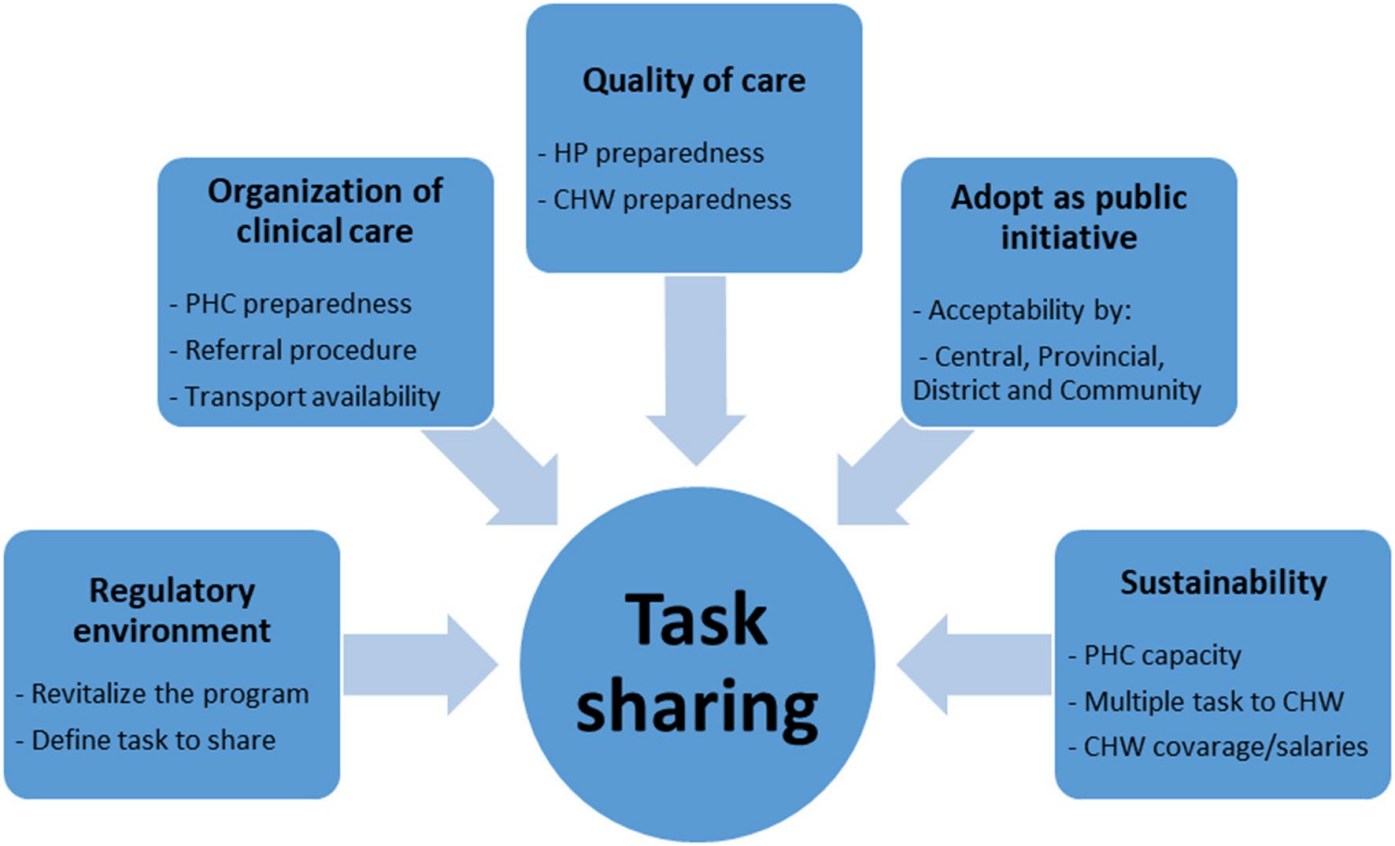
Analysis scheme

Focus group discussions and in-depth interviews were digitally recorded using Olympus AS-2400 PC® recorders. In addition, FGDs, IDIs and the open-ended question from the CHW questionnaire were transcribed verbatim by the same team members who completed data collection. In-depth interviews and the CHW questionnaire were collected and transcribed in Portuguese, while focus group discussions were translated to Portuguese from the local language as needed while being transcribed. Quality control of transcripts was ensured by listening to 25% of the audio recordings and comparing them against the transcripts for accuracy and completeness. The qualitative data were analysed using NVivo version 10.0 (QSR International Pty. Ltd. 2012). A thematic analysis approach was taken (see Fig. 2). The coding structure (based on free nodes, branched nodes, attributes and some pre-determined queries) was developed in advance based on study objectives through a collaborative discussion between researchers at CISM and the University of British Columbia (UBC). Themes were subsequently adjusted, and new themes added as they emerged from the data. As the analysis was performed by two teams (CISM and UBC), the coding structured was in English. The two Mozambican social scientists coded all transcriptions in Portuguese by reading the text in Portuguese and labelling the concepts using the codes in English. Three IDI and two FGD transcripts were translated from Portuguese to English and coded by a social scientist based at UBC for three purposes: first, to support the discussions on the development of the coding structure; second, for the UBC collaborator to be familiar with the raw data, to assist interpretation; and finally, for quality control. To allow the two teams to work independently, the data was split into two NVivo projects, but the same coding structure was used for both teams. Regular coding consensus meetings to discuss data analysis strategy and findings were held via Skype™. The coding agreement between the coders was very high. When the coding was completed, the analysed data were merged into a single project managed by the Mozambican team, from which the final queries, interpretation and data reduction were conducted.

To define our study constructs, we used the 2007 WHO task-sharing global recommendation and guidelines. In this document, we found five essential recommendations to guide task-sharing: adoption as a public health initiative, enabling regulatory environment, ensuring the quality of care, ensuring organization of the clinical care service and ensuring the sustainability [37].

### Ethical considerations

This study received approval from the CISM Institutional Review Board (CIBS_CISM/08/2013) and the University of British Columbia in Canada (H12-00132). All participants in the study provided informed consent after receiving a written study information sheet and a detailed verbal explanation. For the illiterate participants, a literate witness was asked to read and explain to the participant the contents of the participant information sheet. The consent form was signed by the witness, and the researcher assistant after the participant’s fingerprint was taken. All identifiable data of participants were anonymised through attribution of unique identification numbers or pseudonyms to guarantee anonymity. Before the field activities, the first contact was made with the Ministry of Health, the Provincial and District Health Directorate to obtain permission for data collection.

## Results

### Organization of clinical care

The health facility assessment questionnaire was completed by 56 chief nurses and 10 midwives. Fifty-six (56) health facilities were assessed: one quaternary, two tertiary, seven secondary and 46 primary health centres (see Fig. 1). All health facilities assessed were public, managed by the Ministry of Health and offer ANC and delivery services. The entry point to the health system was the PHC, and it was recommended that CHWs refer to this level. All facilities reported at least one type of blood pressure machine (digital or manual) to screen for hypertension and pre-eclampsia. Albumin urine tests (proteinuria) were not available at the primary level and in more than half (57%) of secondary facilities (see Table 1).

**Table 1:** Participants demographic information

Characteristic			CHWs (%) N = 81		Stakeholders (%) N = 8
Age					
20–29			14 (17%)		1 (12%)
30–39			12 (15%)		3 (38%)
40–49			25 (31%)		3 (38%)
> 50			21 (26%)		1 (12%)
Missing			9 (11%)		–
Gender					
Male			20 (25%)		3 (38%)
Female			53 (65%)		5 (62%)
Missing			8 (10%)		–
Marital status					
Married			26 (32%)		3 (38%)
Divorced			3 (4%)		–
Widowed			5 (6%)		–
Single			38 (47%)		5 (62%)
Missing			9 (11%)		
Highest level of education attained					
Primary level uncompleted			39 (48%)		–
Primary level completed			15 (19%)		–
Secondary level uncompleted			14 (17%)		–
Secondary level completed			1 (1%)		4 (50%)
Higher degree			–		4 (50%)
Missing			12 (15%)		
Years of experience as CHW					
1 year			21 (26%)		NA
2 years			15 (19%)		NA
3 years			6 (7%)		NA
> 3 years			29 (36%)		NA
Missing			10 (12%)		NA
Number of CHW interviewed per district					
Province	District	Number of existing CHWs		Number of CHWs interviewed	
Maputo	Manhiça	15		15	
	Magude	27		23	
Gaza	Bilene Macia	14		14	
	Chokwe	5		5	
	Xai-Xai	15		15	
	Chibuto	17		9	

Methyldopa was available in most primary (76%) and secondary (86%) level health facilities. The interviewees said that the availability of antihypertensives depends on what they receive from the central drug store. Even though the guideline recommends hydralazine, methyldopa or nifedipine, prescribing practice reflect availability. MgSO_4_ was available in most primary (83%) and all secondary (100%) level facilities, although stockouts had been reported in one primary, two tertiary and one quaternary facility (see Table 1).

### Quality of care assurance

#### Health facility-based professionals’ preparedness

At the primary level, only one to two nurses with basic (grade 7 + 3 years of nurse training) or moderate training (grade 10 + 2 years of nurse training) were responsible for maternity health, and the numbers of providers remain low in higher levels of care. Midwives were called maternal and child health nurses (MCHN) (*enfermeiras de saúde materno-infantil*), and they were all full-time employees in health facilities. These nurses were responsible for all obstetric care at the primary and secondary level. Some MCHN were trained to perform caesarean sections (see Table 1). Obstetricians were only found in tertiary and quaternary facilities.

All nurses reported awareness of PE/E and had been trained to identify the danger signs of pregnancy, including those related to PE/E. The diagnosis of PE/E was based on symptoms and the measurement of blood pressure without assessing proteinuria. They are trained to administer MgSO_4_ before referral to the nearest higher-level facility. There is an obstetric care guidebook with a chapter on diagnoses and management of PE/E and a flowchart of procedures and algorithms in case of PE/E including drugs to be administered and dosages. This guidebook and flowchart were available in all delivery rooms.

For the treatment of women with pre-eclampsia, the nurses mentioned having methyldopa and hydralazine. In the case of convulsions, nurses reported using magnesium sulphate intravenously before referral.

*I… medications that I have to treat complications… I just have serums, and I have magnesium sulphate, methyldopa, and I have hydralazine. These are the medications that I have here. I usually give methyldopa and hydralazine when a woman has severe pre-eclampsia.* **MCH nurses**

*Uh... [Laughs] Magnesium sulphate … which we usually give when you have convulsions, we just suddenly take a bottle and give (give) it directly into the vein.* **MCH nurses**

#### Community health workers’ preparedness

For this study, 81 CHWs were identified, representing 87% of all CHWs in the study area. The CHWs were from Manhiça (15), Magude (23), Bilene Macia (14), Chokwe (5), Xai-Xai (15) and Chibuto (9) (see Table 2). In Maputo, four CHWs, all from Magude District, were not included because they lived very far from the health facility where data collection occurred or were not reachable at the time of study recruitment. In Gaza, eight CHWs, all from Malehice and Chibuto District, were not included as data collection took place during and immediately after flooding and access to and communications with the health units was not possible. IDIs were also done with three CHW supervisors, three District Chief Medical Officers, and two obstetrician-gynaecologists from AMOG. A total of 26 FGDs were conducted with pregnant women (5), partners and husbands (5), mothers and mothers-in-law (5), matrons and TBAs (5), community leaders (1) and health workers (5). The panel discussion was with four IRB members (1), Community Advisory Board (1), Ministry of Health (1) and Provincial Directorate (1) (see Tables 2 and 3).

**Table 2:** Characteristics of focus group discussion participants

#	Stakeholder Group	Region	# of Participants	Age (Median)	Marital status	Occupation	Schooling level
1	Women of reproductive age	Ilha Josina Machel	9	30	Married (9)	Farmer (9)	Never studied (3) Primary (6)
2		Calanga	3	25	Married (3)	Farmer (3)	Primary (3)
3		Três de Fevereiro	8	23	Married (8)	Farmer (8)	Never studied (1) Primary (4) Secondary (3)
4		Messano	8	28	Married (6) Widow (1) Single (1)	Farmer (7) Nurse (1)	Primary (7) Secondary (1)
5		Chongoene	Unknown*	*Unknown*	*Unknown*	*Unknown*	*Unknown*
6	Mothers and mothers-in-law	Ilha Josina Machel	12	29	Married (7) Widow (5)	Farmer (12)	Never studied (4) Primary (8)
7		Calanga	12	59	Married (7) Widow (3) Divorced (2)	Farmer (12)	Never studied (1) Primary (11)
8		Três de Fevereiro	*Unknown*	*Unknown*	*Unknown*	*Unknown*	*Unknown*
9		Messano	8	39	Married (5) Widow (1) Single (2)	Farmer (8)	Never studied (6) Primary (2)
10		Chongoene	11	46	Married (3) Widow (1) Single (7)	Housewife (11)	Never studied (3) Primary (8)
11	Partners and husbands	Ilha Josina Machel	12	37	Married (12)	Farmer (12)	Never studied (3) Primary (8) Secondary (1)
12		Calanga	4	37	Married (4)	Farmer (1) Fisherman (1) Traditional healer (1) Locality chief (1)	Never studied (1) Primary (3)
13		Três de Fevereiro	10	45	Married (10)	Farmer (3) Security (2) Seller (1) Mason (1) Small jobs (2) Gas station clerck (1)	Primary (10)
14		Messano	6	42	Married (6)	Farmer (6)	Primary (6)
15		Chongoene	7	49	Married (3) Single (4)	Farmer 6) Driver (1)	Primary (7)
16	Matrons and traditional birth attendants	Ilha Josina Machel	6	55	Married (3) Widow (3)	Farmer (6)	Never studied (5) Primary (1)
17		Calanga	9	67	Married (3) Widow (4) Divorced (2)	Farmer (9)	Primary (9)
18		Três de Fevereiro	12	65	Married (4) Widow (7) Divorced (1)	Farmer (12)	Never studied (9) Primary (3)
19		Messano	10	43	Married (5) Widow (3) Single (2)	Farmer (8) Teacher (1) Housewife (1)	Never studied (1) Primary (7) Secondary (2)
20		Chongoene	9	58	Married (2) Widow (1) Single (6)	Housewife (9)	Never studied (7) Primary (2)
21	Health professionals	Ilha Josina Machel	4	28	Married (4)	Health technician (1) Health agent (3)	Primary (1) Secondary (3)
22		Calanga	*Unknown*	*Unknown*	*Unknown*	*Unknown*	*Unknown*
23		Três de Fevereiro	2	51	Married (2)	Midwife (1) Health technician (1)	Secondary (2)
24		Messano	3	26	Married (2) Single (1)	Nurses (2) Counsellor (1)	Secondary (3)
25		Chongoene	2	30	Single (2)	Nurse (1) Assistant (1)	Secondary (2)
Panel discussion							
1	Community Advisor Board	Manhiça	6	57	Married (4) Widow (1) Divorced (1)	community leader and neighborhood chief (4) Traditional healer (1) Activist (1)	Primary (6)
2	IRB	Manhiça	6	39	Married (3) Single (3)	Medical doctor (2) Teacher (1) Judge (1) Theologian (1) Community Liaison Officer (1)	Licenciature (5) Secondary (1)

*Missing data

**Table 3:** Health facility assessment on available resources for diagnose and manage PE/E

Type of facility surveyed	Primary		Secondary		Tertiary		Quaternary		Total	
	N	%	N	%	N	%	N	%	N	%
Total	46		7		2		1		56	
Equipment for PE/E diagnose										
Digital sphygmomanometer	30	65%	2	29%	0	0%	0	0%	32	57%
Manual sphygmomanometer	16	35%	6	86%	2	100%	1	100%	25	45%
Pulse oximeter	0	0%	0	0%	0	0%	1	100%	1	2%
Basic diagnostic test										
Dipstick proteinuria	0	0%	2	29%	2	100%	1	100%	5	9%
Medications for PE/E										
Methyldopa PO	35	76%	6	86%	2	100%	1	100%	44	79%
MgSO_4_	38	83%	7	100%	2	100%	1	100%	48	86%
Referral transportation										
Has a functional ambulance or other vehicle for emergency	2	4%	7	100%	2	100%	1	100%	12	21%
Has transportation for patients referred to another facility	43	93%	7	100%	2	100%	1	100%	53	95%
Has access to an ambulance or other vehicle for emergency transportation to another facility or that operates from another facility	44	96%	7	100%	2	100%	1	100%	54	96%
Health professionals, median (IQR)										
Obstetricians	0	(0–0)	0	(0–0)	5	(1–5)	N/A	N/A	5	(1–5)
Nurse	1	(1–2)	5	(5–8)	24	(17–30)	N/A	N/A	5	(1–4)

CHWs do regular home visits for pregnant women. During these visits, they provide health education about the importance of ANC and facility delivery, and check the ANC record to confirm the pregnant women was attending the scheduled visits. Their visits also include identifying of warning signs in pregnancy: anaemia, oedema, vaginal bleeding, and abdominal pain. According to our questionnaire, most CHWs (94%) agreed that they knew the warning signs of pregnancy, although fewer were familiar with the warning signs of hypertension in pregnancy (41%); despite this, most report recognition of convulsions (70%). The CHWs were not trained nor equipped to detect hypertension and proteinuria. In line with these findings, few were comfortable providing oral medications (47%) or injections (5%) of any kind to pregnant women. The management of specific complications in pregnancy, such as pre-eclampsia and eclampsia, is not part of CHWs’ regular tasks.

According to supervisors, CHWs are skilled to identify the basic danger signs of pregnancy, but they could not manage these emergencies; instead, they refer.

*The CHWs are not trained to identify hypertension in pregnancy. In cases of eclampsia, they immediately refer the pregnant women. As they are not trained nor equipped to manage these situations, one can assume that they do not have an in-depth understanding of the condition and its complexities*. **CHW supervisor**

Matrons and TBAs also attend pregnant women at the community level, but they are discouraged by the health system from assisting deliveries at home.

*“There was [childbirth in the community], but it emerged that they could no longer give birth at home, midwives can no longer work, they [pregnant women] must go to the hospital, and if the hospital is far away, they have to go first and stay at a maternity waiting home. We helped [the pregnant women], but there was a time when they said not to give birth at home, you have to give birth at the facility [at the hospital]”.* **Matron**

*“Yes, we help [women] giving birth, but with the arrival of the hospital, we were forbidden [by the Ministry of Health]. [Women] should go to the hospital. It is not because we cannot do [the] birth. We cannot do it; our time ended up. And now we cannot get involved in these new things [new rules]. Our daughters are brought to the hospital now”.* **TBA**

According to focus group participants, matrons provide advice to women throughout pregnancy, particularly related to traditional practices. The matrons do not formally offer health services, but when there is an emergency in the community, they might be called upon for assistance.

“*We have no work; if they give us work, we will do […]. This order (to visit pregnant women in the houses) we have not had yet but if they give us the order we will visit […].”—***Matron**

#### Acceptability of pre-eclampsia identification and emergency management by CHWs

During the panel discussions, stakeholders from the MoH were favourable to task-sharing to CHWs, and they were involved in efforts to revitalize the CHW programme. However, participants from provincial directorate raised concerns regarding the complexity of curative care on top of the already burdened CHWs. In addition, some were concerned that families would not accept a male CHW to visit a pregnant woman at home.

Obstetrician-gynaecologists were supportive of involving CHWs in community-based interventions in pregnancy; they believed that such interventions would further support maternal and child health nurses. However, they were sceptical of giving CHWs the responsibility of PE/E management, especially regarding the administration of intramuscular MgSO_4_ and the management of potential adverse reactions. They considered only obstetricians-gynaecologists as suitable to evaluate the feasibility or endorse policy related to pregnant women.

*“I want to be in peace with myself and my conscience after recommending something which I know will be good for the patients. If I recommend something that I know will bring problems, I will not feel comfortable”*. **Obstetrician-Gynaecologist**

District medical officers supported CHW involvement, especially identifying and referral of early-stage pre-eclampsia to avoid severe disease, including eclampsia. However, they were concerned regarding CHWs ability to manage cases in the community and were not supportive of the administration of injectables by CHWs.

The CHW supervisors did not raise major concerns regarding the involvement of CHWs in the management and referral of PE/E. Caring for pregnant women at the community level would not constitute a major change in their routine. However, the supervisors were not confident that, they would diagnose and treat pre-eclampsia or eclampsia, with their current training. In their view, CHWs would only recognise warning signs in general and not specific to PE/E and quickly refer.

Nurses were favourable to the introduction of community management of pre-eclampsia by CHWs. They, however, highlighted the need first to address current barriers to care of pregnant women at health facilities, including lack of equipment, shortage of supervisors, and irregular drug availability.

The CISM IRB members were favourable of CHWs visiting pregnant women at home and administering oral medication but had reservations regarding injectable medication administration, considering that CHWs could not monitor adverse events following injections. Furthermore, the Community Advisory Board was supportive as long as it is approved by the government and local leaders.

The CHWs were supportive of the community management of pre-eclampsia and felt that they could take this on with appropriate training. However, they complained about the lack of transport from the community to the health facility, which could affect their ability to refer.

Community members described how this shift in practice would bring CHWs closer to the community and help decrease the problem of PE/E.

*“Yes, we would accept (CHWs) just knowing that this is a person from the hospital... We could go to his house even he could come to our houses we would accept because he helps us.*- **Pregnant women, Manhiça district**

When husbands/partners were asked if they were willing to accept a male CHW to assist pregnant women, they were generally supportive. Therefore, the aim is to provide care to women, similar to their acceptance of male doctors at the health facility.

*“For me, in my opinion, it would be possible to attend [attend pregnant women by male CHW], I would be calm because [even myself] when I go to the hospital I do not expect only to be attended by male staff, a nurse can also attend me. However, that person was positioned by health services to heal both people […] we just know that is a doctor.”—***Partners and husbands, Messano District**

### Sustainability

Although respondents were supportive of the task-sharing, some important challenges were raised. For example, nurses highlighted the need to create capacity at primary health facilities before task-sharing to CHWs, including improving the availability of proteinuria tests and drugs for PE/E management. On the other hand, ministry of health and provincial directorate representatives raised concerns of the multiple tasks for which CHWs are responsible without adequate training. The low number of CHWs and the low subsidies paid was also a constraint to task-sharing (see Table 4). These concerns should be addressed to ensure the long-term sustainability of such a policy.

**Table 4:** Summary of activities to be shared with CHW and perspectives of the study participants

Tasks to be shared	Already on CHW tasks	Community members	CHW	IRB	CAB	Primary level nurses	CHW supervisors	District level officials	Provincial and Central level officials	Obstetrics and gynecology
Advice about the pregnancy and ANC visits	Yes	Yes	Yes	Yes	Yes	Yes	Yes	Yes	Yes	Yes
Advice about pre-eclampsia/eclampsia	No	Yes	Yes	Yes	Yes	Yes	Yes	Yes	Yes	Yes
Measure blood pressure	No	Yes	Yes	Yes	Yes	Yes	Yes	Yes	Yes	Yes
Measure proteinuria	No	Yes	Yes	Yes	Yes	Only if it is available in primary level	Yes	Yes	Yes	Yes
Administer methyldopa	No	Yes	Yes	Yes	Yes	Only if it is available in primary level	No Not able to diagnose and treat	Yes	No Concerned about too many tasks to CHW	Yes
Administer injectable MgSO_4_	No	Yes	Yes Concern about injections	No, concerns regarding not being able to manage the adverse events	Yes	Only if it is available in primary level	No Not able to diagnose and treat	No Concerns about injections	No Concerned about too many tasks to CHW	No Concerned about Injectable
Identify emergency conditions	Yes	Yes	Yes	Yes	Yes	Yes	Yes	Yes	Yes	Yes
Refer for the nearest facility	Yes	Yes Concerns about transport	Yes Concerns about transport	Yes	Yes	Yes	Yes	Yes	Yes	Yes

## Discussion

This study shows availability of MgSO_4_ in almost all facilities; this represents an improvement from previous reports in Mozambique, where most facilities did not have MgSO_4,_ and the nurses reported low confidence in its administration [38]. However, MgSO_4_ is still not available in 17% of the primary level facilities, like in many African countries; therefore, many women are not receiving this life-saving drug [39]. In addition, the measurement of proteinuria was not part of regular clinical assessment at the primary level and was unavailable in most secondary level facilities [40].

Strengthening the health system with the provision of the necessary equipment and medication for the prevention and treatment of PE/E is key to reducing of maternal mortality and facilitating task-sharing between nurses and CHWs. The need for health system strengthening has also been reported for other maternal intervention such as the prevention of neonatal sepsis [41].

Nurses reported having been trained and equipped to measure blood pressure, identify the danger signs of pregnancy, use MgSO_4_ pre-referral, and contact an ambulance to transport critical patients. However, shortage of health professionals and distance to health facilities were described as challenges for obstetric care where task-sharing with CHWs has been proposed. Although nurses reported being trained and equipped to manage PE/E, most primary level facilities only have one or two nurses dedicated to maternal care. This aligns with the minimal availability of trained and qualified health workers, with an average of 0.087 physicians and 0.526 nurses and midwives per 1000 population in the country, far short of the WHO recommendation of one nurse and one doctor/1000 population [27]. A study that aimed to evaluate the magnitude of the health facility-based maternal mortality and assessed the health facility factors implicated in these deaths was conducted in 2008 in Mozambique. This study showed that the risk of maternal death was reduced by 40% in health facilities with nurses trained in maternal and child health and able to provide emergency obstetric care (EmOC). The study has proven the high impact of the mid-level skilled maternal and child health nurses on reducing maternal mortality [42]. There is a need to improve the availability of skilled professionals to ensure the quality of care.

In this study, nurses complained that there were barriers to providing health care at health facilities, which included the lack of equipment, lack of supervision and the irregular availability of medicines. This finding was also found in a recent study that reported that the availability of sexual and reproductive health commodities in health facilities was low in Kenya, Tanzania, Uganda and Zambia [43]. These barriers must be resolved before task-sharing efforts will be successful.

To mitigate the shortfall in the number of health professional, Mozambique adopted a CHW program strategy to improve access to care. In addition, the revitalization of the program has been an opportunity to introduce new community-level interventions. This study also reported that the CHW were confident to include additional tasks in their duties. They were well respected and accepted by their communities which can contribute to improved adherence to new interventions. The district medical officers and CHW supervisors complained about the low level of training, the low number of CHW available in the study area and their low subsidies. These results are similar to other studies in Mozambique reporting that apart from their low level of education and training, the limited number of CHWs, the lack of supervision, and their low subsidies are challenges to task-sharing with this cadre [44–47].

Training on the detection of danger sign in pregnancy could facilitate the identification of pre-eclampsia and eclampsia by CHWs; however, the provision of an injectable (MgSO_4_) medication was a barrier to the management of pre-eclampsia by CHWs as it is not part of their regular training. As a result, medical officers and specialists believe that the CHWs are not prepared to identify and manage pregnancy complications with injectable MgSO_4_.

A previous study in Mozambique showed that Ninety-three percent of CHW had an awareness of pregnancy complications. In cases of eclampsia, CHWs reported referring the women immediately. However, the vast majority of the CHWs surveyed said they could neither measure blood pressure nor proteinuria (90%). In addition, fewer reported confidence in providing oral antihypertensives (14%) or injections in pregnancy (5%) [35]. These results illustrate the limited knowledge of the community providers in Mozambique and the need for enhanced regular training. Increasing evidence suggests that CHWs can provide additional services after adequate training, including prevention of post-partum haemorrhage with misoprostol [48, 49] and injectable contraception with Depo-Provera [50, 51]. Involvement of the District medical officers and OBGYNs in CHWs task-sharing initiatives, including identifying PE/E cases and managing with MgSO_4_ before referral, could increase the intervention uptake. However, studies to assess the CHW’s ability to administer PE/E interventions are needed before task-sharing.

CHWs were confident delivering more interventions, and the community also accepted their role. The study showed that CHWs are well received in their communities and felt empowered with new interventions. Gender preference of CHWs was explored in this study; however, it was not found to be a concern. Nevertheless, this should be interpreted with caution as it may not be generalizable across the country [47]. The concern of CHW regarding lack of transport from the community to the health facility was aligned with what was reported by the community. This challenge needs to be addressed urgently' as the intervention is pointless if the women are unable to reach the health facilities for further management of the condition. However, in the context of delays in transportation, commencing treatment of eclampsia sooner though the CHWs may cause less harm than if MgSO_4_ was only be administered at the health facility [14]. Further research demonstrating the ability of CHWs to implement complex intervention would increase the acceptability of the task-sharing by MoH and gynaecologist.

### Limitations

Facility assessment reflects one point in time and does not represent what is available at all time. The availability of equipment, for example, the blood pressure machine, does not indicate that the nurses are using it correctly for PE/E diagnosis. The qualitative data were collected in four communities in Maputo and Gaza Provinces; although these areas show a good representation of the region, results may not be generalizable to other context than the areas involved in this study. Due to the translation of the data (between *Changana*, Portuguese and English), some subtleties of meanings may have been lost; however, strict quality control steps were put in place throughout the transcription, translation and coding processes to minimize this limitation.

We could not interview all CHWs in the study area; some were the hardest to reach, and we lost the opportunity to have their thoughts. However, other CHW from the same location were included, which minimized the risk of affecting our results.

The decision to not involve TBAs in the intervention trial led to miss information regarding the acceptability of this group to the proposed task-sharing.

## Conclusions

This study showed that task-sharing of initial screening and pre-referral management of pre-eclampsia and eclampsia is deemed feasible and acceptable by the community. But an effort should be in place to remove barriers at the health system level. There is a need to mitigate stakeholder perceptions of CHWs’ inability for complex tasks. Studies to assess CHWs’ ability to administrate PE/E intervention are needed before implementing task-sharing.

## Data Availability

The datasets used and analysed during this study will be stored at the CISM repository and are available by request to the corresponding author after adhering to the CISM policy on data sharing.

## Abbreviations

AMOG: Associação Moçambicana de Ginecologistas
ANC: Antenatal care
CHW: Community health workers
CISM: Centro de Investigação em Saúde da Manhiça
CLIP: Community level interventions for pre-eclampsia
FGD: Focus group discussions
FIGO: International federation of gynaecologists and obstetricians
HIV: Human immunodeficiency virus
iCCM: Integrated community case management
IDI: In-depth interviews
LMICs: Low and middle-income countries
MgSO_4_: Magnesium sulphate
MMR: Maternal mortality rate
PE/E: Pre-eclampsia/eclampsia
PHC: Primary health care
UNICEF: United Nations Children’s Fund
WHO: World Health Organization
